# TIPdb-3D: the three-dimensional structure database of phytochemicals from Taiwan indigenous plants

**DOI:** 10.1093/database/bau055

**Published:** 2014-06-13

**Authors:** Chun-Wei Tung, Ying-Chi Lin, Hsun-Shuo Chang, Chia-Chi Wang, Ih-Sheng Chen, Jhao-Liang Jheng, Jih-Heng Li

**Affiliations:** ^1^School of Pharmacy, Kaohsiung Medical University, Kaohsiung 80708, Taiwan, ^2^PhD Program in Toxicology, Kaohsiung Medical University, Kaohsiung 80708, Taiwan and ^3^National Environmental Health Research Center, National Health Research Institutes, Miaoli County 35053, Taiwan

## Abstract

The rich indigenous and endemic plants in Taiwan serve as a resourceful bank for biologically active phytochemicals. Based on our TIPdb database curating bioactive phytochemicals from Taiwan indigenous plants, this study presents a three-dimensional (3D) chemical structure database named TIPdb-3D to support the discovery of novel pharmacologically active compounds. The Merck Molecular Force Field (MMFF94) was used to generate 3D structures of phytochemicals in TIPdb. The 3D structures could facilitate the analysis of 3D quantitative structure–activity relationship, the exploration of chemical space and the identification of potential pharmacologically active compounds using protein–ligand docking.

**Database URL**: http://cwtung.kmu.edu.tw/tipdb.

## Introduction

Plants have been valuable resources of inspirations for the development of therapeutic agents (1–2). It was estimated that current global market for plant-derived drugs is worth >20 billion, and the market continues growing. However, only 10–15% of plant species have been explored for developing clinically important drugs (3). Taiwan is located at the boundary of tropical and subtropical areas with a broad range of altitude. The isolation of the islands from continent further contributes to the abundance of endemic species in Taiwan. Owing to the unique geographical features and location, Taiwan is rich in diversity of plants (4). The wide diversity of plants in Taiwan and their associated phytochemicals, which are evolved as part of the plant defense system in response to environmental stress (5), provides a great opportunity for the discovery of novel pharmacologically active compounds. To facilitate the drug discovery process, we had created a manually curated TIPdb database collecting published anticancer, antiplatelet and antituberculosis phytochemicals with their associated activity information from indigenous plants in Taiwan (6).

The first release of TIPdb contains 99 indigenous plants in Taiwan with >5000 bioactivity records of phytochemical compounds and their two-dimensional (2D) structures (6). A comparison of TIPdb with existing databases of plant natural products has been reviewed elsewhere (7). TIPdb curating taxonomy, bioactivity and 2D structure information is useful for exploring novel chemical spaces and developing quantitative structure–activity relationship (QSAR) models. To further assist the structure-based virtual screening of novel bioactive compounds, three-dimensional (3D) chemical structures are required for applications of protein–ligand docking and 3D-QSAR. As a successful example, the application of structure-based virtual screening led to the identification of novel inhibitors against *Mycobacterium tuberculosis* L-alanine dehydrogenase (8). With the wealthy collection of bioactive phytochemical compounds, the construction of 3D structure database for TIPdb is valuable for drug discovery.

Recently, several useful 3D structure databases of natural products have been developed, including TCM database@Taiwan (9), KNApSAcK-3D (10), 3DMET (11) and NuBBE database (12). The TCM database@Taiwan focused on natural compounds from traditional Chinese medicine. Nakamura *et al*. converted the 2D structures of plant metabolites from KNApSAcK (13) to construct a 3D structure database KNApSAcK-3D. The 3DMET is a 3D structure database constructed by converting 2D chemical structures of Kyoto Encyclopedia of Genes and Genomes (KEGG) COMPOUND collection (14). NuBBE database curated natural products from the biodiversity of Brazil. The construction of TIPdb-3D structure database containing numerous unique phytochemicals could largely help the exploration of the chemical space of natural products and structure-based virtual screening.

The previous work of TIPdb focused on the curation of bioactive phytochemicals of Taiwan indigenous plants from published literatures. To provide a comprehensive 3D database of phytochemicals from Taiwan indigenous plants, the TIPdb has been expanded to cover all plants listed in Flora of Taiwan, second edition (4). The 3D structures in TIPdb-3D are either generated from 2D structures using the MMFF94 force field or extracted from KNApSAcK-3D database. Currently, there are more than 8800 non-redundant 3D structures of phytochemicals associated with 1116 Taiwan indigenous plants. Additionally, >5200 compounds are identified to meet drug-like properties based on the Lipinski’s rule of five (15). TIPdb-3D is thereby expected to be a useful resource for natural product research.

## Construction

The development of TIPdb-3D contained two parts: the conversion of 2D structures from existing chemicals in TIPdb to 3D structures, and the collection of 3D structures from the existing 3D structure databases of KNApSAcK-3D.

For the generation of 3D structures, two softwares of Balloon (16–17) and DG-AMMOS (18) were used. The Balloon software based on a multi-objective genetic algorithm was firstly used to convert 2D structures in TIPdb to 3D structures for maintaining a high compatibility to 3D structures collected from KNApSAcK-3D. Briefly, 300 generations were used to search for chemical 3D structures with the lowest energy.

For those chemicals that Balloon failed to generate 3D structures, DG-AMMOS was applied to the conversion of 3D structures, and hydrogen atoms were subsequently added by using Open Babel (19). The 3D conversion of DG-AMMOS is based on a molecular simulation package AMMP (20). Both softwares, Balloon and DG-AMMOS, used MMFF94 (Merk Molecular Force Field) (21) to calculate the energies of chemicals.

For the collection of 3D structures from the existing 3D structure database of natural products, a full list of Taiwan indigenous plants was at first collected from the Flora of Taiwan, second edition (4). The full names of Taiwan indigenous plants were subsequently applied to query KNApSAcK-3D for retrieving corresponding 3D structures of phytochemicals with references.

MySQL server edition 5.1 was used to implement TIPdb-3D. The web interface and all functions were implemented using PHP, HTML and JavaScript languages. Jmol applet of version 13.0 (22) was used to interactively display chemical 3D structures.

Drug-like compounds are favorable for drug discovery. To identify the subset of drug-like compounds from TIPdb-3D, the drug-like properties of the chemical structures were analyzed using the Lipinski’s rule of five. The Lipinski’s rule of five defines four criteria by analyzing the physicochemical properties of >2000 drugs (15): First, the molecular weight is <500 Dalton. Second, the octanol– water partition coefficient log*P* is <5. Third, the number of hydrogen bond donors is <5. Fourth, the number of hydrogen bond acceptors is <10. The PaDEL-descriptor (23), a software for calculation of molecular descriptors and fingerprints based on the Chemistry Development Kit (24), is used to calculate the properties and violations of Lipinski’s rule of five.

## Content and Utility

There are a total of 4077 indigenous plants listed in Flora of Taiwan, second edition (4). Despite extensive searches of published literatures and databases, only less than half Taiwan indigenous plants have been researched for their phytochemicals. Currently, the TIPdb-3D database contains the 3D structures of a total of 8853 non-redundant chemicals from 1116 Taiwan indigenous plants that have been curated into the database. In addition, there are 13 173 records of chemical–plant associations available in TIPdb-3D.

In the process of 3D structure conversion by the Balloon software, only four chemicals failed to be converted. The four chemicals were then successfully converted to 3D structures by the DG-AMMOS software. Among the four chemicals, three chemicals of TIP002275, TIP002276 and TIP002117 belong to the class of triterpenoid, and the chemical of TIP002031 is a flavonoid. The conversion of 1794 3D structures took 6.5 h on a computer equipped with an AMD FX-8120 eight-core processor (3.1 GHz) and 32GB RAM.

**Figure 1.**
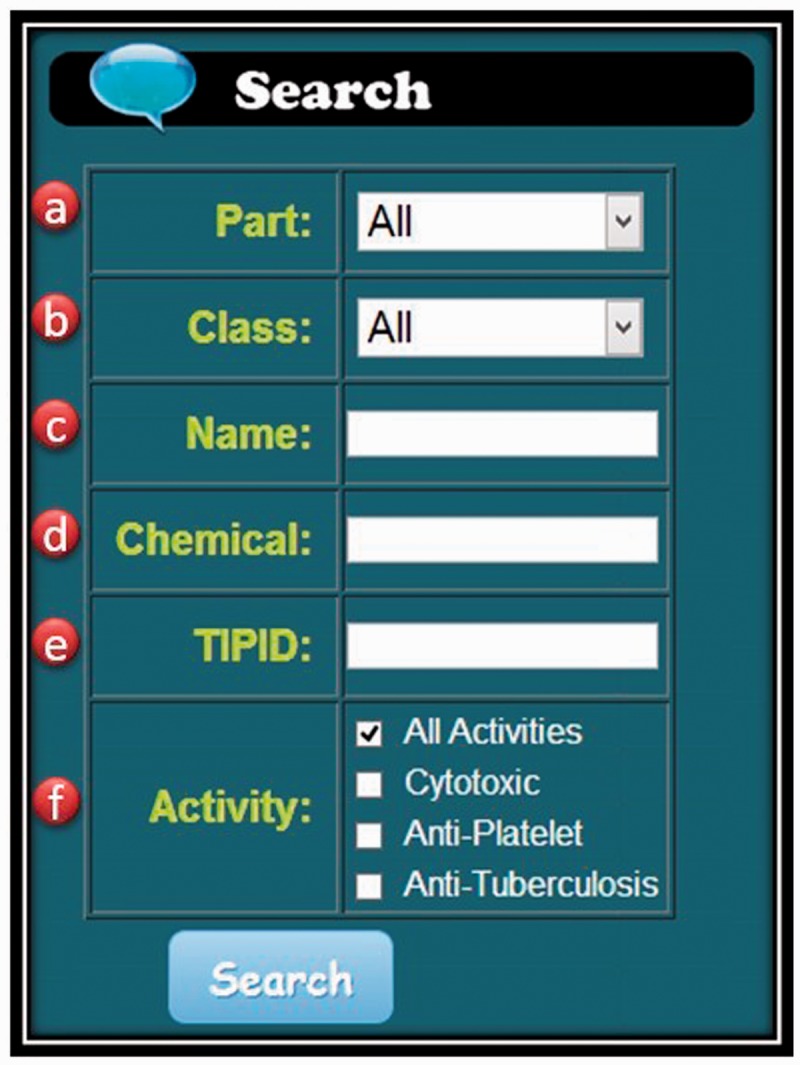
The search function. Users can search TIPdb-3D database by the following keywords: (**a**) part of a plant; (**b**) class of a chemical; (**c**) botanical name of a plant; (**d**) chemical; (**e**) TIPID; and (**f**) bioactivity.

**Figure 2.**
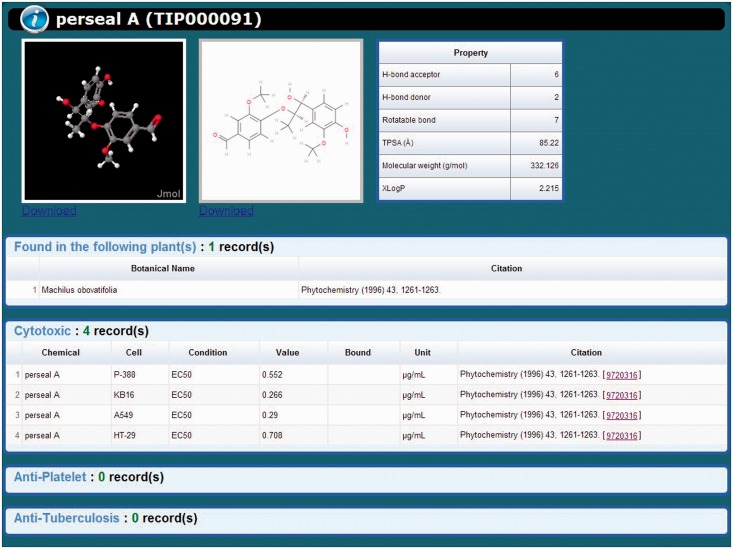
An illustrated record of TIPdb-3D. The TIPID is shown in the parenthesis after the chemical name. A typical record consists of 3D and 2D structures, physicochemical properties, associated plant(s) and bioactivities.

The TIPdb-3D database has been fully integrated with TIPdb, which is equipped with both taxonomy browsing and search functions. The taxonomy tool enables the browsing of Taiwan indigenous plants from 63 orders, 195 families, 680 genus and 1116 species. The search function has also been improved to provide the chemical TIPID search function as shown in Figure 1. A typical record of a chemical in TIPdb-3D is shown in Figure 2, containing a 3D structure, a 2D figure, physicochemical properties, associated plants and its cytotoxicity, antiplatelet and antituberculosis bioactivity data. Both the 2D figure and 3D structure files are downloadable for each chemical using the browsing tool and search function. Six physicochemical properties are available in the database for each chemical, including—(i) the number of hydrogen-bond acceptors; (ii) the number of hydrogen-bond donors; (iii) the number of rotatable bonds; (iv) topological polar surface area (TPSA); (v) molecular weight; and (vi) XLogP. The integrated Jmol applet of version 13.0 enables the interactive display of 3D structures.

**Figure 3.**
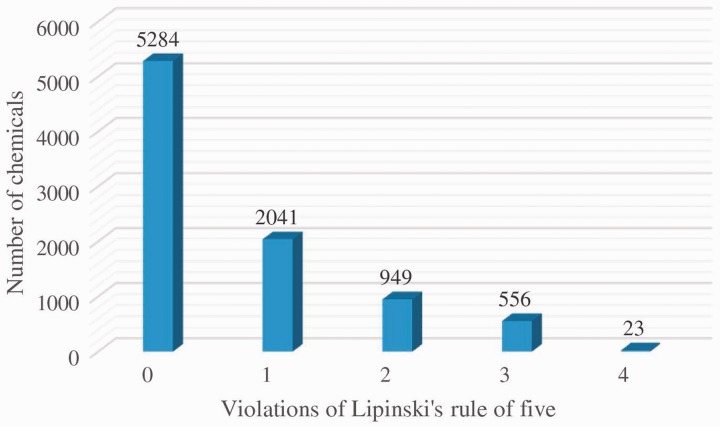
The distribution of chemicals with drug-like properties.

To identify potential drug candidates, the drug-like filter of Lipinski’s rule of five was applied to 8853 chemicals. Figure 3 shows the distribution of chemicals with various numbers of rule violations. A total of 5284 chemicals with no violation of Lipinski’s rule of five are identified as potential drug candidates. The numbers of chemicals with one, two, three and four rule violations are 2041, 949, 556 and 23, respectively. We select chemicals without any rule violation to create a drug-like chemical data set of 3D structures for structure-based virtual screening. The whole sets of all the chemical 3D structures and drug-like chemical 3D structures are downloadable from TIPdb-3D Web site (http://cwtung.kmu.edu.tw/tipdb/download.php) as the Structure Data Format.

## Discussion

This study presents a useful database, TIPdb-3D, with 3D structures of phytochemicals from Taiwan indigenous plants that has been integrated into TIPdb. Among the 8853 non-redundant chemicals, >1500 phytochemicals from TIPdb-3D have not been included in other phytochemical databases with chemical–plant associations. The unique contents of TIPdb-3D could complement other databases and enable the exploration of chemical space diversity. In addition, drug-like chemicals that pass Lipinski’s rule of five were identified and made downloadable for drug discovery. The most distinct feature of TIPdb-3D is its curation of both bioactivities and 3D chemical structures. TIPdb-3D is expected to be a valuable resource for the analysis of 3D-QSAR and structure-based virtual screening of bioactive compounds for anticancer, antiplatelet and antituberculosis activities.

The database has been under active development to collect more phytochemicals with structures and bioactivities from published literatures. In addition to the cytotoxicity, antiplatelet and antituberculosis available in TIPdb, more bioactivities such as anti-inflammatory are being curated. To provide better insights into bioactivities of chemicals, future works include the integration of target and bioactivity information from chemical–protein interaction and chemical bioactivity databases such as STITCH (25), Comparative Toxicogenomics Database (26) and CARLSBAD (27).

## Funding

National Science Council of Taiwan [NSC 101-2311-B-037-001-MY2]; Kaohsiung Medical University Research Foundation [KMU-M103009]; NSYSU-KMU Joint Research Project [NSYSUKMU103-P002]; National Health Research Institutes [EH-103-PP-09]. Funding for open access charge: Kaohsiung Medical University Research Foundation [KMU-M103009].

*Conflict of interest*. None declared.
